# Resveratrol induces apoptosis by modulating the reciprocal crosstalk between p53 and Sirt-1 in the CRC tumor microenvironment

**DOI:** 10.3389/fimmu.2023.1225530

**Published:** 2023-07-27

**Authors:** Aranka Brockmueller, Constanze Buhrmann, Parviz Shayan, Mehdi Shakibaei

**Affiliations:** ^1^ Chair of Vegetative Anatomy, Institute of Anatomy, Faculty of Medicine, Ludwig-Maximilians-University Munich, Munich, Germany; ^2^ Institute of Anatomy and Cell Biology, Faculty of Medicine, University of Augsburg, Augsburg, Germany; ^3^ Department of Parasitology, Faculty of Veterinary Medicine, University of Tehran, Tehran, Iran

**Keywords:** colorectal cancer, tumor microenvironment, resveratrol, apoptosis, Sirt-1 modulation, p53, caspase-3, reciprocal crosstalk

## Abstract

**Introduction:**

P53 represents a key player in apoptosis-induction in cancers including colorectal cancer (CRC) that ranks third worldwide in cancer prevalence as well as mortality statistics. Although a pro-apoptotic effect of resveratrol has been repeatedly proven in CRC cells, its pathway mechanisms are not completely understood, as there are controversial statements in the literature regarding its activation or inhibition of the counteracting proteins Sirt-1 and p53.

**Methods:**

CRC cells as wild-type (HCT-116 WT) or p53-deficient (HCT-116 p53^-/-^) were cultured using multicellular tumor microenvironment (TME) cultures containing T-lymphocytes and fibroblasts to elucidate the role of p53/Sirt-1 modulation in resveratrol’s concentration-dependent, pro-apoptotic, and thus anti-cancer effects.

**Results:**

Resveratrol dose-dependently inhibited viability, proliferation, plasticity as well as migration, and induced apoptosis in HCT-116 WT more effectively than in HCT-116 p53^-/-^ cells. Moreover, resveratrol stimulated Sirt-1 expression when administered at low concentrations (<5µM) but suppressed it when added at high concentrations (>10µM) to CRC-TME. In parallel, similar to the knockdown of Sirt-1 at the mRNA level, treatment with high-concentration resveratrol boosted the acetylation of p53, the expression of p21, Bax, cytochrome C, caspase-3, and ultimately induced apoptosis in CRC WT but not in CRC p53^-/-^ cells. Notably, increasing concentrations of resveratrol were found to promote hyperacetylation of p53 and FOXO3a as post-translational substrates of Sirt-1, indicating a negative regulatory loop between Sirt-1 and p53.

**Discussion:**

These results demonstrate for the first time, a negative reciprocal crosstalk between the regulatory circuits of p53 and Sirt-1, consequently, apoptosis induction by higher resveratrol concentrations in CRC-TME.

## Graphical Abstract

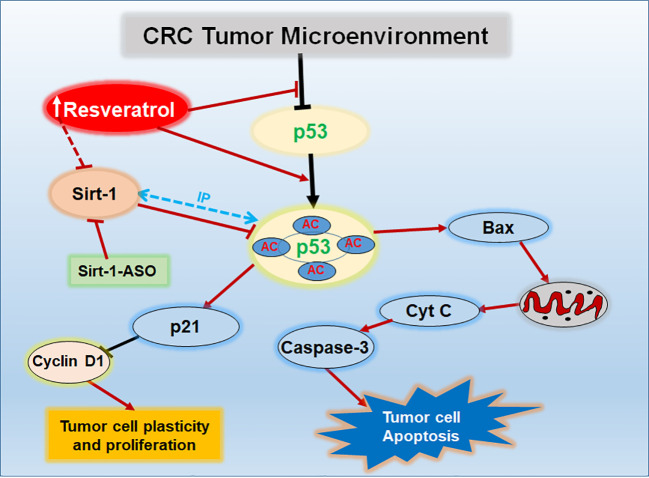

## 1 Introduction

Cancer is already one of the leading causes of death worldwide and is expected to become the leading cause of death in almost every country over the course of this century (1). One of the most common malignant neoplasms with increasing prevalence is colorectal cancer (CRC). Its development is influenced not only by advancing age but also significantly by the modern human lifestyle. Meanwhile, it is known that a high number of cases occurs mainly in population groups with a lower level of education or unhealthy lifestyle (2).

For effective treatment, it is crucial not only to delay the disease, but to eliminate cancer cells in the long term to enable recovery and at the same time prevent recurrence. A central element of apoptosis initiation is the p53 tumor protein (cellular tumor antigen p53), which is also referred to as the “guardian of the genome” due to its enormous importance in key cellular processes such as cell apoptosis, autophagy, division, genome stability, immune response and regulation of the tumor microenvironment (TME) (3). At a healthy cell status, p53 is bound to the murine double minute 2 gene (mdm-2) oncogene that suppresses its activation (4). However, in the case of severe deoxyribonucleic acid (DNA) damage, molecular cascades enable the activation of p53, overcoming its inhibition and inducing apoptosis (5). Interestingly, many different tumors take advantage of this scenario. Consequently, a massive proliferation of CRC cells is made possible, among other things, by relatively low p53 expression (6). Although classical chemotherapeutics such as 5-fluorouracil (5-FU), cisplatin, and mitomycin C indirectly raise the p53 level (7), they are not direct modulators of this pathway and CRC’s p53-status seems not to be a determinant for significant treatment success with these drugs (8). Therefore, it is of great interest to find multisystem modulators that could have a more targeted effect on p53-regulation and complement the therapy of CRC patients without additional side effects.

In this respect, phytopharmaceuticals are a practicable possibility, and among them in particular the natural polyphenol resveratrol, which naturally occurs in many edible plants such as grapevine, cranberries, and peanuts (9–11). In addition to its known anti-inflammatory, anti-microbial, and anti-oxidative effects (12), this phytoalexin also has an anti-carcinogenic impact in numerous cancer types: breast tumors, prostate cancer, and lung carcinoma (13–15) to name a few examples. Particularly in CRC cells, resveratrol develops extensive cancer-inhibiting effects. Contextually, the phytopharmaceutical acts as an anti-inflammatory (16), anti-proliferative (17, 18), anti-plasticity, and anti-metastatic (18, 19) as well as a pro-apoptotic (20, 21). On this occasion, silent information regulator 1 (Sirt-1) is considered a central intracellular target of resveratrol in different CRC cell lines such as LoVo, SW480, and HCT-116 (22, 23). Among mammals, there are seven sirtuins (Sirt-1 - 7); Sirt-1, Sirt-6, and Sirt-7 are primarily found in the nucleus, Sirt-3, Sirt-4, and Sirt-5 in the mitochondria, and Sirt-2 in the cytosol. Sirtuins were initially characterized as deacetylases, however, a larger category of functions is now being identified (24).

Sirt-1 represents a type III nicotinamide adenine dinucleotide (NAD^+^)-dependent histone/protein deacetylase (HDAC) with a regulating impact on several cellular processes and especially repair of DNA damages (25). While Sirt-1 expression is up-regulated in normal colon tissue or benign colon polyps, a significant Sirt-1 down-regulation in CRC has already been proven (26). It seems to be a double-edged sword, since on the one hand Sirt-1 enables the longevity of normal cells, but at the same time, it plays a special role in tumorigenesis. This fact is mainly due to a deacetylation and thus blocking apoptosis-related p53 and Forkhead box O3a (FOXO3a) protein (27–29). FOXO3a is a part of the FOXO transcription factor family and forms an intracellular unity with Sirt-1 to ensure resistance to oxidative stress. Their interaction initiates cell cycle regulation but simultaneously prevents the initiation of apoptosis (28, 29). An opposite process, confirming that a down-regulation of Sirt-1 enables an up-regulation of p53 acetylation, which subsequently entails apoptotic cascades around the p21/cyclin D1-axis as well as the caspase-3/-9 signaling is often portrayed (30). Nevertheless, various research works have demonstrated an up-regulation of Sirt-1 in CRC cells by resveratrol (low concentrated), and in parallel, apoptosis pathways were induced (22, 23). Overall, there are indications in the current literature that both Sirt-1 (22) and p53 (31) are among the key targets of resveratrol to unfold its anti-CRC effects.

We became aware of these partially contradictory statements and hypothesized a switch in targeting Sirt-1/p53 signaling depending on resveratrol concentration. With this present work, we illuminate a gap in CRC science by focusing on resveratrol’s regulation of a negative reciprocal relationship between the regulatory pathways of p53 and Sirt-1, and consequently, apoptosis induction by higher resveratrol concentrations in CRC-TME. For our study, we used HCT-116 CRC cells, more precisely a comparison between their wild type (HCT-116 WT) and those with a p53-deficiency (HCT-116 p53^-/-^). The experiments were carried out in 3D-culture models *in vitro* in order to simulate the multicellular TME mimicking *in vivo*-like characteristics.

## 2 Materials and methods

### 2.1 Antibodies and reagents

The following materials were purchased from Sigma-Aldrich (Taufkirchen, Germany): resveratrol, alginate, MTT reagent, 2-mercaptothanol, DAPI, and antibody against β-actin (MAB #A4700). Anti-p53 (MAB #sc-126), anti-Bax (MAB #sc-7480), anti-cytochrome C (MAB #sc-13156) and normal IgG (mouse/rabbit) were from Santa Cruz (CA, USA), while anti-caspase-3 (PAB #AF835) and anti-cyclin D1 (MAB #MAB4314) were from R&D Systems (Heidelberg, Germany). Antibodies against Sirt-1 (MAB ab191385, PAB ab12193) or FOXO3a (MAB ab240127, PAB ab70315) were obtained from Abcam (Berlin, Germany). Anti-acetyl-lysine antibody (MAB ac-k-103) was from Cell Signaling Technology (Beverly, MA, USA). Secondary sheep anti-mouse and sheep anti-rabbit alkaline phosphatase-linked antibodies were from Millipore/Merck Chemicals (Darmstadt, Germany), and rhodamine-coupled secondary antibodies were purchased from Dianova (Hamburg, Germany). Resveratrol was prepared as 100mM stock solution and further diluted in the cell culture medium during the experiments without exceeding an ethanol concentration of 0.1%.

### 2.2 Cell lines and cultivation

Human HCT-116 WT (CRC cells) and human MRC-5 (fibroblasts) were from the European Collection of Cell Cultures (Salisbury, UK). Human p53-deficient HCT-116 p53^-/-^ (CRC cells) were a generous gift from Prof. Dr. Ajay Goel (CA, USA). Furthermore, human Jurkat (T-lymphocytes) were bought at the Leibniz Institute (Braunschweig, Germany). HCT-116 WT, HCT-116 p53^-/-,^ and MRC-5 were grown adherently, while Jurkat cells were grown non-adherently in T175 cell culture flasks from Thermo Fisher Scientific (Schwerte, Germany). All cells were cultivated at 5% CO2, at 37°C and in cell culture medium from Sigma-Aldrich (Taufkirchen, Germany): DMEM F-12 (1:1), supplemented with 10% fetal bovine serum (FBS), 1,2% penicillin/streptomycin, 1% L-glutamine, 1% ascorbic acid, 1% essential amino acids and 0,5% amphotericin B as used before (19).

### 2.3 Sirt-1 knockdown

Transient transfection for the purpose of Sirt-1 knockdown was implemented by antisense oligonucleotides (ASO) whose sequence was 5′-GTATTCCACATGAAACAGACA-3′. The sense oligonucleotides (SO) with a 5′-TGTCTGTTTCATGTGGAATAC-3′ sequence functioned as a control. Both substances were purchased from Eurofins (Ebersberg, Germany). During the experiments, the CRC cells were transfected by a suspension of 0.5µM Sirt-1-ASO or Sirt-1-SO in 10µl Invitrogen Lipofectin Transfection Reagent (Fisher Scientific, Schwerte, Germany) per ml cell culture medium containing 3% FBS. This method has been used previously (22).

### 2.4 CRC alginate beads

Alginate beads were produced as previously published (17, 32). In short, CRC cells (HCT-116 WT or HCT-116 p53^-/-^) were counted and resuspended (1 million/ml) in 2% alginate in an 0.15M NaCl solution. This composed suspension was polymerised drop by drop for 10 minutes in a CaCl_2_ solution. Then a triple wash in Hanks salt solution and a double wash in cell culture medium (10% FBS) were carried out, followed by incubation in cell culture medium (10% FBS) for 30 minutes. CRC cells were cultivated in alginate beads for 10-14 days, and the change of cell culture medium (3% FBS) and treatment additives were carried out every other day. The investigations were carried out without TME (basal control) or with TME (MRC-5, Jurkat).

### 2.5 Multicellular tumor microenvironment (TME)

The TME is a multicellular composition to simulate the conditions of a human body affected by cancer without animal testing. For this purpose, a 3D environment was created in well-plates, in which a monolayer of fibroblasts grew on the bottom and T-lymphocytes floated in the cell culture medium. The CRC cells (HCT-116 WT or HCT-116 p53^-/-^) were either embedded into alginate beads and added to the TME or grown as monolayers on glass plates and placed on a small steel bridge into the TME. Both variations were already well established (17, 33).

### 2.6 MTT method

The MTT method was used to determine the CRC cells’ viability. After 10-14 cultivation days, HCT-116 WT or HCT-116 p53^-/-^ cells were dissolved from alginate as already extensively described (17). Afterward, the CRC cells were resuspended in MTT medium (without vitamin C/phenol red) and pipetted into a 96-well-plate under the addition of MTT solution. After three hours, the reaction was stopped by MTT solubilization solution, and evaluation was carried out at 550nm optical density (OD) with an Elisa Reader from Bio-Rad (Munich, Germany).

### 2.7 Western blotting

Western blotting was performed to determine the expression levels of diverse proteins. Therefore, HCT-116 WT or HCT-116 p53^-/-^ were cultivated in alginate beads for 10-14 days. After test completion and alginate bead sampling, the CRC cells were dissolved from the alginate matrix as explained in-depth (17). The ensuing HCT-116 WT or HCT-116 p53^-/-^ cells were treated with a lysis buffer, followed by centrifugation (30 minutes, 4°C, 10.000RPM) and supernatants were frozen at -80°C. The sample preparation was carried out with a Protein Quantification Kit from Interchim (Montlucon Cedex, France) and 2-mercaptoethanol. A transblot apparatus from Bio-Rad (Munich, Germany) was used for Western blotting (SDS-PAGE) as previously described (33). In short, pre-incubated nitrocellulose membranes (Fisher Scientific, Schwerte, Germany) were incubated in a primary antibody (1:10.000, overnight), followed by incubation with a secondary antibody (1:10.000, 90 minutes). A program (Quantity One, Bio-Rad, Munich, Germany) was used for densitometric analysis.

### 2.8 Immunoprecipitation

Immunoprecipitation served to represent a functional connection between two proteins. Firstly, CRC cells were prepared as Western blot samples, precleared with normal mouse or rabbit IgG, and by incubation in Staphylococcus aureus. Secondly, the samples were processed with a primary antibody for two hours and incubated with Staphylococcus aureus again for one hour. The precise procedure as well as SDS-PAGE separation by Western blotting was widely described (34, 35).

### 2.9 Phase contrast/Immunofluorescence investigation

For phase contrast or immune-fluorescence investigation, HCT-116 WT or HCT-116 p53^-/-^ cells were cultivated on small round cover-glasses. After approximately 60% confluent growth, the cover-glasses were transferred into 6-well-plates containing a small steel net bridge. In this 3D culture model, they were treated with or without TME, were left to grow another day, and then treated for two hours (as stated in the figures). Afterward, CRC cells on cover-glasses were evaluated in one of the two following ways: (A) Observation by phase contrast microscope (Zeiss Axiovert 40 CFL from Oberkochen, Germany) and photographic documentation as explained in (19). The number of CRC cells was calculated by counting five microscopic fields per culture. (B) Fixation in methanol and subsequently frozen at -20°C. Afterward, immunolabeling was performed: Defrosting, washing with Hanks salt solution, incubation in Triton solution and bovine serum albumin solution. Then, incubation with primary antibody (1:80, overnight, moist chamber) as well as secondary antibody (1:100, 90 minutes, moist chamber), staining with DAPI (15 minutes, well-plate) and covering in Fluoromount. The evaluation of the CRC-cover-glasses was carried out by Leica DM2000 (Wetzlar, Germany) microscope and photographs were digitally stored. This whole process has already been published earlier (17, 22).

### 2.10 Wound migration

To perform a CRC wound migration assay, HCT-116 WT or HCT-116 p53^-/-^ cells were also grown on small round cover-glasses for 24 hours. Then, the CRC monolayer was divided per incision (representing a wound) with an Eppendorf (Hamburg, Germany) pipette tip. Thereafter, the cover-glasses were gently rinsed with Hanks salt solution, incubated in cell culture medium, and photographed with an Axiovert 40CFL microscope from Zeiss (Oberkochen, Germany). After two hours of treatment, the CRC-cover-glasses were placed into the 3D culture, as described in ‘Immunofluorescence investigation’. On day three, the CRC-cover-glasses were located in 12-well-plates to observe the wounds and after two more days of 3D cultivation, they were rinsed with Hanks again, photographed, fixed in methanol, frozen at -20°C and later immunolabelled as described above.

### 2.11 Transmission electron microscopy (TEM)

The ultrastructural morphology of HCT-116 WT or HCT-116 p53^-/-^ cells was investigated with a TEM 10 from Zeiss (Jena, Germany). After cultivation of the CRC cells as described in ‘Immunofluorescence investigation’, the CRC-cover-glasses were fixed in Karnovsky solution for one hour, transferred into tubes with a cell scraper, and fixed in osmium tetroxide for two hours. The subsequent dehydration was performed by an ascending alcohol series. Then, samples were embedded in Epon, processed with Reichert-Jung Ultracut E (Darmstadt, Germany), and contrasted as explained in (20).

### 2.12 Statistics

All experiments were repeated three times and all data were statistically considered by student’s t-test and one-way ANOVA (*post hoc*) to bring out the differences of parameters in each group. After determining fundamentally significant differences in a simple analysis of variance, more detailed direct comparisons were made by *post hoc* analysis to show which mean values ​​differ significantly. In this relation, percentage effects and 95% confidence intervals were determined. The statistical significance was set at p<0.05 and expressed as means ± standard deviations.

## 3 Results

At the core of the present study was the switching of Sirt-1 activity as an intracellular target of resveratrol and deacetylase for p53 signaling-dependent apoptosis in response to higher resveratrol concentrations. For our study, we used HCT-116 CRC cells of wild type (HCT-116 WT) and those lacking p53 (HCT-116 p53^-/-^). Experiments were performed in 3D culture models *in vitro* to simulate the multicellular TME, which has similar characteristics as *in vivo*.

### 3.1 Resveratrol represses CRC cell viability significantly more effectively in HCT-116 WT than in HCT-116 p53^-/-^ cells

**Figure 1:**
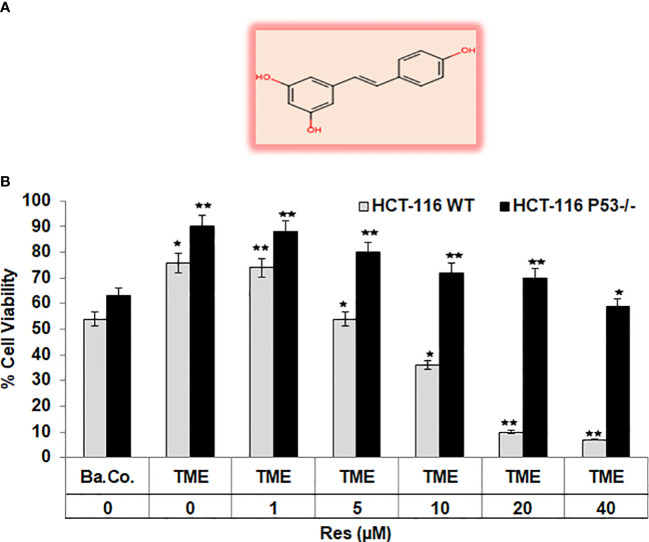
Resveratrol’s impact on CRC cell viability. **(A)** Chemical structure of *trans*-resveratrol. **(B)** HCT-116 WT or HCT-116 p53^-/-^ cells were cultivated in alginate beads, then isolated and their viability was measured by MTT assay. X-axis shows the treatments: basal control (Ba.Co., without TME, without resveratrol), TME control (with MRC-5, Jurkat, without resveratrol), or TME with resveratrol (1, 5, 10, 20, or 40µM Res). Y-axis shows the number of viable CRC cells, measured at 550nm. Grey bars represent HCT-116 WT cells, while black bars represent HCT-116 p53^-/-^ cells. Values related to TME control: *p<0.05, **p<0.01.

To get a first assessment of resveratrol’s anti-CRC effect in a p53-dependent manner, resveratrol’s concentration-dependent (0, 1, 5, 10, 20, 40µM) impact on HCT-116 WT or HCT-116 p53^-/-^ cells in 3D alginate beads was compared by MTT viability test. Initially, a significant increase in CRC cell viability by TME (including fibroblasts and T-lymphocytes) was confirmed as earlier published (17, 33). Compared with a basal control, the TME constellation enhanced the number of viable cells by more than 30% in both CRC cell lines (**Figure 1**). An addition of 1µM resveratrol to CRC-TME did not cause significant differences in HCT-116 WT or HCT-116 p53^-/-^ cells, but from addition of 5µM resveratrol, p53-deficiency became noticeable. In HCT-116 WT cells, resveratrol exerts a distinct viability-limiting effect, which increases with rising concentrations. The viability of HCT-116 WT cells was significantly reduced by 3%, 29%, 53%, and 90% by treatment with 1, 5, 10, 20, and 40µM resveratrol, respectively, compared to the TME control (**Figure 1**). These observations differ strongly from those in HCT-116 p53^-/-^ cells, where the effect of resveratrol on cell viability was much weaker. Here, 5µM of the phytopharmaceutical restricted the viability by 15% while 20µM reduced it by less than 30%, and even at 40µM resveratrol treatment, 60% of CRC cells survived compared to TME control (**Figure 1**). Altogether, resveratrol is able to suppress the viability of HCT-116 WT cells more effectively than the viability of HCT-116 p53^-/-^ cells leading to a first indication that p53 might play an important role in resveratrol’s proliferation-inhibition and the mechanisms could be concentration-dependent.

### 3.2 Resveratrol reduces CRC cell plasticity and proliferation significantly more effectively in HCT-116 WT than in HCT-116 p53^-/-^ cells

**Figure 2:**
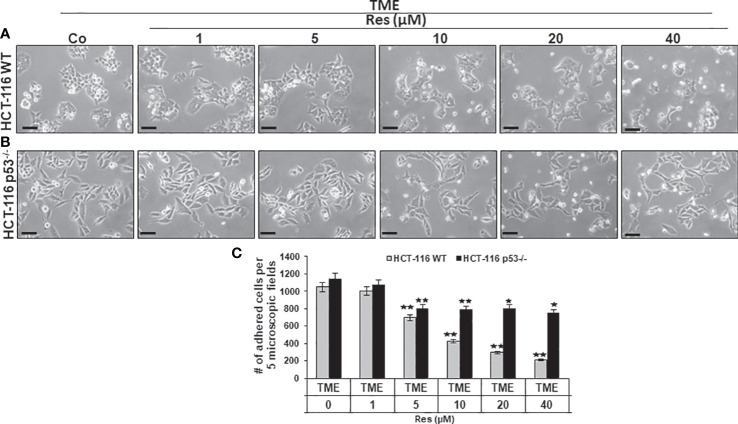
Impact of resveratrol and role of Sirt-1 on CRC cell proliferation. HCT-116 WT **(A)** or HCT-116 p53^-/-^ **(B)** cells were grown on small cover glasses in a 3D culture environment, treated differently, and photographed (phase contrast, x400 magnification). Scale bar corresponds to 30nm. **(C)** illustrates statistic evaluation. X-axis shows the treatments: TME control or TME with 1, 5, 10, 20, 40µM resveratrol. Y-axis shows cell numbers of adhered CRC cells. They were calculated by counting 5 microscopic fields per culture. Grey bars represent HCT-116 WT cells, and black bars represent HCT-116 p53^-/-^ cells. Compared to TME control: *p<0.05, **p<0.01.

Next, focusing on CRC cell proliferation behavior, HCT-116 WT or HCT-116 p53^-/-^ cells were sown as a monolayer on small cover-glasses and placed in a 3D culture environment. Besides a TME control, the previously described treatments (1-40µM resveratrol) were examined and then evaluated by phase contrast microscopy. In HCT-116 WT cells, resveratrol’s addition had striking concentration-dependent effects on a) their proliferation capacity as well as b) their morphology. Related to TME control, a treatment with 1µM did not result in significant changes and at 5µM resveratrol, nearly two-thirds of proliferated HCT-116 WT cells were still counted. This concentration-dependent decrease continued and while 10µM resveratrol suppressed half of the proliferation rate, only 20% of HCT-116 WT cells were adhered at 40µM resveratrol treatment compared to TME control (**Figures 2A, C**). The higher the resveratrol concentration, the fewer migrating pseudopodia were seen, especially above 10µM, which changed the appearance of mesenchymal plasticity. Further, the cells developed a rounded, epithelial-looking character, especially from 20µM onwards (**Figures 2A, C**). In accordance with the MTT results, a treatment with resveratrol effected much fewer changes in HCT-116 p53^-/-^ cells than in CRC WT cells. Compared to TME control, an addition of 1µM resveratrol was barely noticed. Furthermore, when more resveratrol was added, 65-70% of HCT-116 p53^-/-^ cells remained adhered, regardless of the concentration, which was varied between 5µM and 40µM (**Figures 2B, C**). The statistical evaluation showed an overall comparable inhibition of proliferation by 40µM resveratrol in HCT-116 p53^-/-^ cells as by 5µM resveratrol in HCT-116 WT cells (**Figure 2C**). Overall, resveratrol inhibited cell plasticity by inducing a round shape only in HCT-116 WT cells, resulting in cells detaching from the culture dish, but not in HCT-116 p53^-/-^ cells, suggesting that resveratrol at high concentrations induces cell death in p53-dependent signaling.

### 3.3 Resveratrol causes concentration-dependent nuclear Sirt-1-down-regulation and simultaneously p53-up-regulation in CRC cells

**Figure 3:**
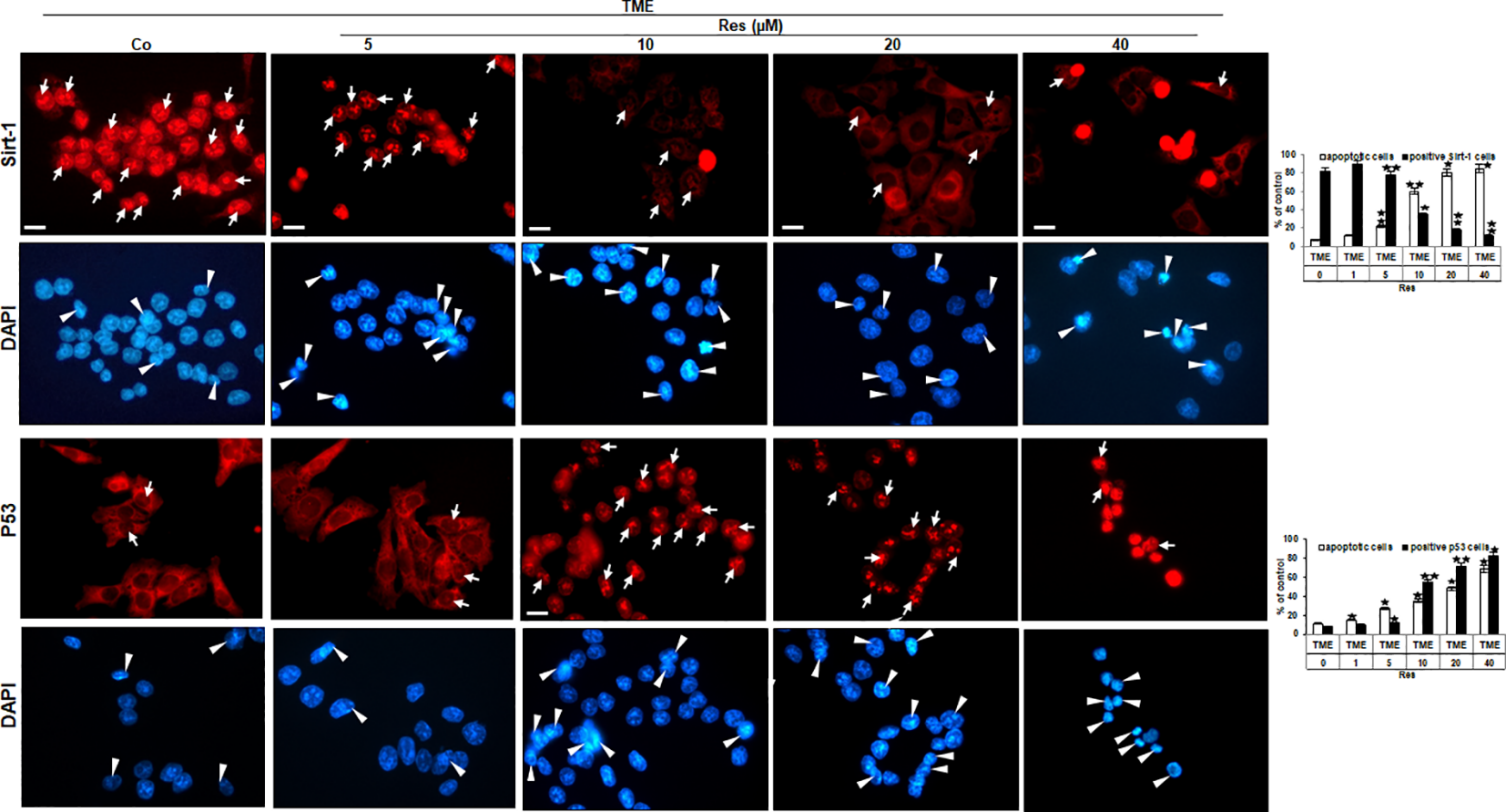
Resveratrol’s impact on nuclear Sirt-1 or p53 expression in CRC cells. HCT-116 WT cells were grown on glass coverslips, treated differently (TME control (Co) or TME with 5, 10, 20, 40µM resveratrol), and afterward immunolabeled against Sirt-1 (red, row 1) or p53 (red, row 3) and stained with DAPI (blue, row 2 and row 4). White arrows mark immunolabelling, and white arrowheads mark apoptosis. Additionally, statistics were applied by counting 5 microscopic fields each. X-axis: treatments. Y-axis: apoptotic CRC cells (white bars) or positive immunolabeled CRC cells (black bars), stated in %. Values: *p<0.05, **p<0.01, in reference to TME control.

The findings of phase contrast observation combined with known knowledge of the central importance of Sirt-1 in resveratrol’s anti-CRC effects (22, 23) led to an interest in examining Sirt-1 expression in HCT-116 WT cells. Therefore, CRC cells were sown on cover-glasses, integrated into a 3D TME, and processed as TME control (without resveratrol) or treated with resveratrol (5, 10, 20, or 40µM) immunolabelled with anti-Sirt-1 antibody and evaluated *via* immunofluorescence microscopy. The CRC cells in TME control showed a very pronounced Sirt-1 marking in their nuclei, which has been somewhat mitigated by the addition of 5µM resveratrol. At 10µM resveratrol, the Sirt-1 immunolabelling was clearly less frequent and strongly attenuated, and from a concentration of 20µM resveratrol, hardly any Sirt-1 positive labeling was observed (**Figure 3**). In parallel, the HCT-116 WT cells were stained with DAPI providing their vitality and indicating apoptotic changes. Here, with increasing resveratrol concentration, an increasing amount of apoptosis was noticeable (**Figure 3**). With this in mind, we decided to investigate HCT-116 WT cells for nuclear expression of apoptosis-associated p53 (3) using the same concentrations as described. As expected, the CRC cells of TME control were very active and showed rarely p53 marking which hardly changed at 5µM resveratrol either. However, if the HCT-116 WT cells were treated with 10µM or 20µM resveratrol, nuclear p53 expression was significantly up-regulated and reached a maximum at 40µM resveratrol addition (**Figure 3**). Consistent with and confirming this, the number of apoptosis increased with increasing resveratrol application, as did the strength of the immunolabelling (**Figure 3**). In total, this observation led us to assume an opposing regulation of Sirt-1 and p53 based on concentration-dependent modulation by resveratrol. Remarkably, it should be noted that resveratrol activates Sirt-1 only at low concentrations, but inhibits the enzyme at high concentrations. At the same time, this allows activation of p53 at high resveratrol concentrations and thus a resveratrol-induced, p53-dependent apoptosis initiation.

### 3.4 Resveratrol suppresses ultrastructural plasticity and induces apoptosis significantly more effectively in HCT-116 WT cells than in HCT-116 p53^-/-^ cells

**Figure 4:**
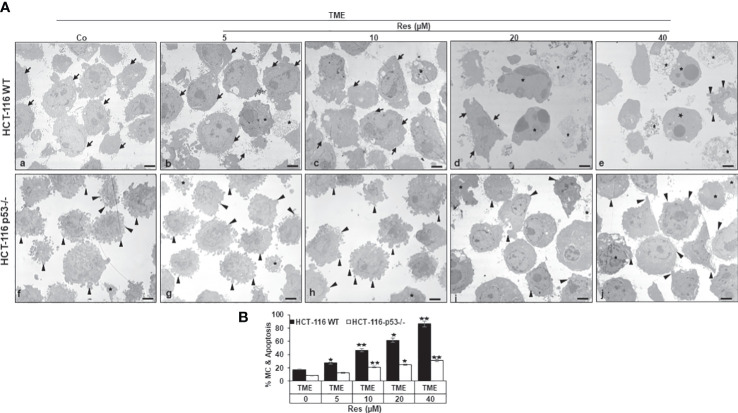
Resveratrol’s impact on CRC cell plasticity. **(A)** HCT-116 WT (a-e) or HCT-116 p53^-/-^ (f-j) cells were grown on small cover-glasses in 3D TME as treatment-free control or enriched with 5, 10, 20, or 40µM resveratrol and then evaluated by transmission electron microscopy (TEM). Scale bars: 1µm, black arrowheads: active pseudopodia, black arrows: epithelial cell surface, black stars: apoptotic bodies. **(B)** X-axis: treatments, black bars: HCT-116 WT cells, white bars: HCT-116 p53^-/-^ cells. Y-axis: mitochondrial changes (MC) and apoptosis in % by counting 5 microscopic fields. *p<0.05, **p<0.01, relative to TME control.

Subsequently, we investigated the effects of resveratrol on CRC cell plasticity at the ultrastructural level, as tumor cell plasticity including their epithelial-mesenchymal transition (EMT) correlates with cancer development and metastasis (36). Taking the role of p53 into account, HCT-116 WT or HCT-116 p53^-/-^ cells were cultured on cover glasses in 3D TME (without treatment or with 5, 10, 20, or 40µM resveratrol) and then evaluated by transmission electron microscopy (TEM). In the overall comparison, it was noticeable that HCT-116 p53^-/-^ cells (**Figures 4Af-j, B**) were more changed by mesenchymal phenotypic plasticity than HCT-116 WT cells (**Figures 4Aa-e, B**), represented by an abundance of pseudopodia and leading to a more aggressive character at the p53-knockdown situation. These differences in the CRC cell surfaces were particularly clear in TME control (**Figures 4Aa,f, B**). With the increasing addition of resveratrol (5-40µM), fewer and fewer pseudopodia were visible, and the CRC cell surfaces became increasingly smooth and thus epithelial. Especially, the number and size of apoptotic bodies including mitochondrial changes increased particularly markedly. Indeed, about 18% of HCT-116 WT cells were apoptotic in the TME control, but at 40µM resveratrol, 85% had mitochondrial changes or already apoptotic bodies (**Figures 4Aa-e, B**). In contrast, HCT-116 p53^-/-^ cells retained their mesenchymal plasticity including distinctive pseudopodia despite resveratrol application. These pseudopodia were still observed after the addition of 20µM or 40µM resveratrol, although they occurred less frequently and far less frequently. Moreover, there were also mitochondrial changes and apoptotic bodies which, however, did not make up more than a third of the HCT-116 p53^-/-^ cells, even at high resveratrol concentrations (**Figure 4f-j**). To sum up, resveratrol’s influences on the phenotypic plasticity of CRC cells were more effective in HCT-116 WT than in HCT-116 p53^-/-^ cells indicating a possible p53-dependent regulation and a wide transcriptional heterogeneity, especially in the case of high-concentrated resveratrol treatment.

### 3.5 Resveratrol suppresses CRC cell migration significantly more effective in HCT-116 WT than in HCT-116 p53^-/-^ cells, comparable to knock-down of Sirt-1 with ASO

**Figure 5:**
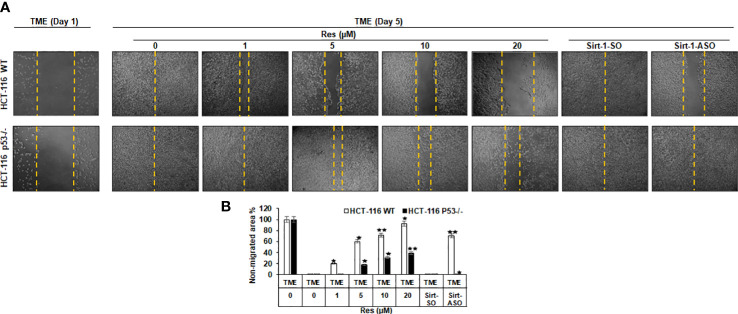
Resveratrol’s impact on CRC cell migration. **(A)** HCT-116 WT (upper row) or HCT-116 p53^-/-^ (lower row) were cultivated in 3D TME and their migration on a wound incision after 5 days was compared. The first column shows a fresh wound on day 1. Further columns show selected treatments: TME control and TME with resveratrol addition (1, 5, 10, 20µM) or Sirt-1-SO/ASO addition (0.5µM). **(B)** Statistic evaluation compares the non-migrated glass area (in %) after 5 days, measured related to fresh incision on day 1. The non-migrated area is marked by yellow dashed lines. In comparison with TME control: *p<0.05, **p<0.01.

Investigation of the effects of resveratrol on the invasion and plasticity behavior of CRC cells considering a possible involvement of Sirt-1 or p53 signaling, HCT-116 WT or HCT-116 p53^-/-^ cells were sown on cover glasses, and the intensity of migration within 5 days after a wound incision was documented. Thereby, CRC cells from both cell lines were left treatment-free (TME control) or treated with resveratrol (1, 5, 10, or 20µM) or transfected with 0.5µM Sirt-1-ASO (Sirt-1 knockdown substance) or 0.5µM Sirt-1-SO (control substance). As shown in **Figure 5**, both cell lines initially grew in a loose monolayer that was evenly incised at day 1 (both rows) and completely overgrown in HCT-116 WT (upper row) as well as HCT-116 p53^-/-^ (lower row) at day 5. In HCT-116 WT cells, it was clearly visible that the free cover-glass area became larger and larger with increasing resveratrol concentration, where the non-migrated area was from 20% (at 1µM) up to 93% at 20µM resveratrol. Completely contradictory, HCT-116 p53^-/-^ cells grew much stronger despite the same resveratrol concentrations so that even with high concentrations of resveratrol (20µM) less than 40% of the incision area remained cell-free (**Figure 5**). Moreover, transient transfection of CRC cells with Sirt-1-SO or Sirt-1-ASO did not restrict the growth of HCT-116 p53^-/-^ cells, and at Sirt-1-SO transfection, the migration of HCT-116 WT cells also remained unaffected. But when HCT-116 WT cells were transfected with Sirt-1-ASO, a gap in the monolayer (70% non-migrated area) was observed after 5 days suggesting resveratrol concentrations from 10µM as alternative Sirt-1-inhibitor (**Figure 5**). Overall, resveratrol acts as a natural Sirt-1 inhibitor with comparable efficacy to the direct silencing of Sirt-1 at the mRNA level by oligonucleotides and suppresses CRC cell migration and plasticity, especially at higher concentrations, at least in part in a p53-dependent manner.

### 3.6 Resveratrol concentration-dependent decreases Sirt-1 signaling, enhances p53 acetylation and cleaved caspase-3 enabling apoptosis-induction in CRC cells

**Figure 6:**
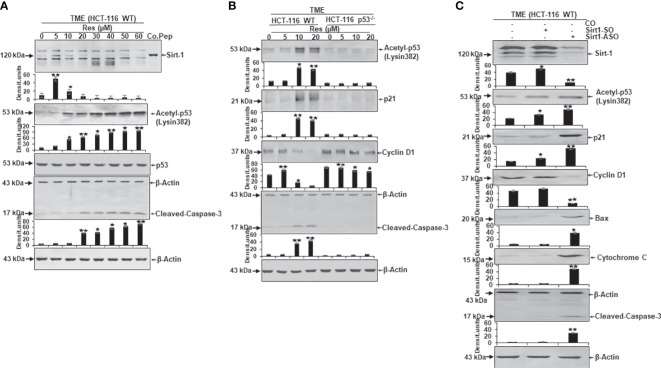
Resveratrol’s impact on apoptosis-related protein expression in CRC cells. HCT-116 WT or HCT-116 p53^-/-^ cells were detached from alginate beads and the expression of apoptosis-associated proteins was investigated per Western Blot. **(A)** HCT-116 WT cells grown as TME control or TME with supplementation of resveratrol (5, 10, 20, 30, 40, 50, 60µM Res). The concentration-dependent effect of resveratrol on Sirt-1, acetyl-p53/Lysin382, p53, cleaved-caspase-3, or β-actin (loading control) was investigated. Furthermore, a control peptide was used that verified Sirt-1. **(B)** HCT-116 WT or HCT-116 p53^-/-^ cells were processed as TME control or enriched with resveratrol (5, 10, 20µM Res), and its concentration-dependent impact on p53, p21, cyclin D1, cleaved-caspase-3 or β-actin (housekeeping protein control) expression was tested. **(C)** HCT-116 WT cells were left treatment-free or transfected with 0.5µM Sirt-1-SO (control substance) or 0.5µM Sirt-1-ASO (knockdown substance). Effects of Sirt-1 knockdown on the expression of Sirt-1, acetyl-p53/Lysin382, p21, cyclin D1, Bax, cytochrome C, cleaved-caspase-3, and β-actin (loading control) were investigated. *p<0.05, **p<0.01.

A further assessment of the dependence of resveratrol’s anti-CRC as well as pro-apoptotic effect from Sirt-1/p53 signaling led to an analysis of protein expression level. Here, CRC cells were embedded in 3D alginate beads, treated in TME, and investigated by Western blotting. Firstly, a series of increasing resveratrol concentrations (0-60µM) were added to HCT-116 WT cells. Here, compared to TME control, resveratrol up-regulated Sirt-1 expression when it was supplemented at low concentration (5µM). But the same phytopharmaceutical demonstrated a significant Sirt-1 expression inhibition when applied in high (greater or equal 10µM) concentrations. A control peptide ensured the correct detection of the Sirt-1 band at 120kDa (**Figure 6A**). Interestingly, the apoptosis-coordinator p53, whose activated form corresponds to acetyl-p53/lysin382, was low expressed in TME control as well as in HCT-116 WT cells that were treated with low-concentrated (5µM) resveratrol. But as soon as the CRC cells were treated with higher resveratrol (greater or equal 10µM) concentration, which was accompanied by Sirt-1-inactivation, in contrast, the expression of acetylated p53 strongly increased (**Figure 6A**). Appropriate thereto, apoptosis-related and p53-triggered cleaved-caspase-3 was clearly up-regulated according to the same dynamics (**Figure 6A**). The even expression of non-acetylated p53, as well as β-actin, served as sample verification (**Figure 6A**).

Secondly, a direct comparison between the expression pattern of HCT-116 WT and HCT-116 p53^-/-^ cells under resveratrol treatment (0-20µM) was carried out. In accordance with **Figure 6A**, the apoptosis-relevant acetylated p53 was low detected in HCT-116 WT cells without treatment or under 5µM resveratrol addition, but activated and hyperacetylated when greater or equal 10µM resveratrol was added to TME cultures. In HCT-116 p53^-/-^ cells, p53 was down-regulated (**Figure 6B**) underlining the aggressiveness as well as apoptosis-resistance of p53-deficient-cells. These changes in the central regulator p53 were also noticeable in further downstream signaling cascades linked to it. Precisely, the protein p21 showed its direct connection with p53 by following its expression dynamics and p21 confirmed its function as an inhibitor of cyclin-dependent kinases which became tangible when the cyclin D1 level was examined (**Figure 6B**). In HCT-116 WT cells, the metastasis-promoting cyclin D1 protein was highly expressed but decreased due to supplementation of high-dose resveratrol (greater or equal to 10µM). However, in HCT-116 p53^-/-^ cells, cyclin D1 was strongly expressed despite treatment with the same resveratrol concentrations (**Figure 6B**). In contrast, cleaved-caspase-3, another major representative of apoptosis, was poorly expressed in HCT-116 WT TME control or TME despite 5µM of resveratrol addition, but this protein was significantly up-regulated by the addition of 10µM or 20µM resveratrol. Interestingly, cleaved-caspase-3 was only marginally found in HCT-116 p53^-/-^ cells regardless of the resveratrol treatment, proving the functional link between p53 and cleaved-caspase-3 (**Figure 6B**). The uniformly displayed β-actin was used as loading control (**Figure 6B**).

Thirdly, to verify whether the Sirt-1 protein affects the acetylation of p53 and is involved in resveratrol-induced activation of p53 as an important intracellular target of resveratrol, we transfected the cells with Sirt-1-SO and -ASO. HCT-116 WT cells were observed as TME control or TME after transfection with 0.5µM Sirt-1-SO/ASO where Sirt-1 knockdown had a significant impact on protein expression levels. Besides the confirmation of Sirt-1-ASO as a knockdown substance, the oppositely regulated interplay between Sirt-1 and p53, which was already noted in **Figures 3**, **6A**, was confirmed (**Figure 6C**). A knockdown of Sirt-1 allowed acetylation and thus activation of p53 as a result of which p21 was also up-regulated and a decrease in the metastatic tendency per repression of cyclin D1 was shown (**Figure 6C**). On the contrary, p53-cofactor Bax, mitochondrial protein cytochrome C, and cleaved-caspase-3 were significantly induced as a consequence of Sirt-1 knockdown compared to TME control or Sirt-1-SO control. These results are consistent with the effects of p53 on cell signaling explained in **Figure 6B** as well as resveratrol- and p53-dependent mitochondrial changes demonstrated in **Figure 4** (**Figure 6C**). Summarized, these Western blot evaluations showed: A) An opposite modulation of the Sirt-1/p53 axis exists with the consequence that resveratrol down-regulates Sirt-1 at high concentrations and thereby paves the way for a p53 activation and the initiation of apoptosis. B) The direct comparison of HCT-116 WT with HCT-116 p53^-/-^ cells reinforces the assumption of a p53-dependent apoptosis-induction by resveratrol. C) An examination of HCT-116 WT cells with Sirt-1 knockdown confirms an opposite expression of Sirt-1 or apoptosis-relevant proteins in CRC cells and underlines the importance of high-concentrated resveratrol as Sirt-1-inhibitor.

### 3.7 Up-regulation of lysine acetylation of Sirt-1 substrates (FOXO3a, p53) and negative functional interplay between the Sirt-1/p53 regulatory cycle by resveratrol in CRC cells

**Figure 7:**
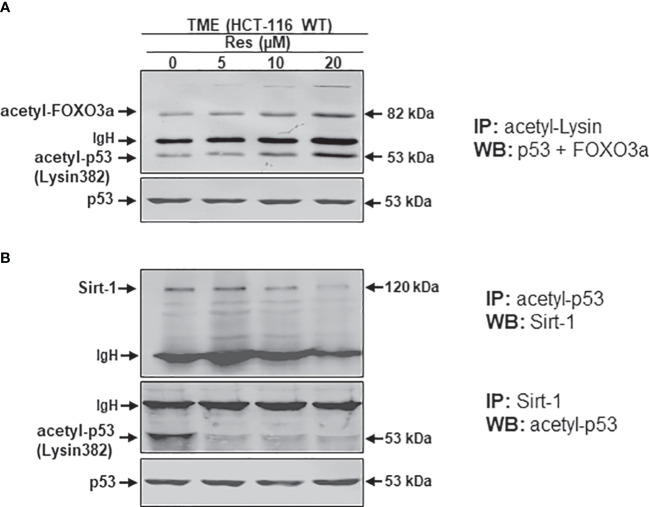
Resveratrol’s impact on p53/FOXO3a acetylation and p53/Sirt-1 negative functional interaction. HCT-116 WT cells were isolated from alginate beads, which were left untreated in TME or treated with resveratrol (5, 10, 20µM Res). Western Blot samples were generated from it and immunoprecipitated (IP) with anti-acetyl-lysin **(A)**, anti-acetyl-p53 or anti-Sirt-1 **(B)**. Then, immunoblotting against acetylated p53 and FOXO3a **(A)** and Sirt-1 or acetylated p53 **(B)** was performed to elucidate their functional connections. IgH means immunoglobulin heavy chain. Non-acetylated p53 verified the CRC samples.

It has been reported that an important intracellular target protein of resveratrol is Sirt-1 and it is activated by resveratrol in various cells (37–39). To finally follow our hypothesis of resveratrol’s apoptosis initiation *via* concentration-dependent Sirt-1/p53 modulation, HCT-116 cells were detached from alginate beads after treating them in TME without additives or combined with 5, 10, or 20µM resveratrol. Subsequently, the CRC samples were immunoprecipitated and processed by Western blotting. Against the background that both, onco-suppressor p53 as well as stress-repressor FOXO3a, are activated by acetylation, and are known as post-translational substrates of deacetylating enzyme Sirt-1 (27, 28, 40). Therefore, and to confirm our previous assumption of Sirt-1 down-regulation by high-dose resveratrol, HCT-116 WT cells were initially immunoprecipitated with anti-acetyl-lysin antibodies and investigated on their p53 and FOXO3a expression after treatment with different resveratrol concentrations (**Figure 7A**). Both Sirt-1 substrates were barely detected in the TME control and despite the addition of low-concentrated resveratrol (5µM) or with increasing concentration (10-20µM) of resveratrol, acetylated p53 and FOXO3a were more and more strongly expressed (**Figure 7A**). Altogether, both proteins showed a dynamic during resveratrol treatment whereby, to the best of our knowledge, the levels of both proteins could be displayed on a common membrane for the first time. The activation of p53 and FOXO3a, accompanied by acetylation, at high-dosed resveratrol, proved the phytopharmaceutical to be an inhibitor of deacetylases and thus Sirt-1 (**Figure 7A**). After this certainty, samples from the same HCT-116 WT cell treatments were immunoprecipitated with antibodies against p53 and Sirt-1 and then immunoblotted against each other. Here, p53-immunoprecipitated CRC cells showed a clear Sirt-1 expression in TME control as well as at low concentrated resveratrol (5µM) treatment. However, this Sirt-1 expression was significantly attenuated by the addition of a higher concentration of resveratrol (10 or 20µM), indicating that Sirt-1 was able to functionally bind to p53 at a low dose of resveratrol but not at a high dose of resveratrol, underscoring the fact that the phytopharmaceutical acts as a natural Sirt-1 repressor above 10µM (**Figure 7B**). When Sirt-1 was immunoprecipitated from the CRC cell samples, the differences became even clearer. Here, acetylated p53 was co-immunoprecipitated with Sirt-1 only significantly expressed in the TME control. A resveratrol supplementation to HCT-116 WT cells led to a repression of co-immunoprecipitation between Sirt-1 and p53, and as a consequence, p53 connection was inhibited even at 5µM resveratrol addition (**Figure 7B**) demonstrating a lost of p53-binding in the course of Sirt-1 down-regulation by rising concentration of the natural polyphenol. All things considered, to the best of our knowledge, these results present for the first time extensive evidence of resveratrol’s concentration-dependent negative Sirt-1/p53 counter-regulation. In this context, these results demonstrate that apoptosis induction of resveratrol at high concentrations (equal to or higher than 10µM) is mediated by negative two-way crosstalk between the regulatory circuits of Sirt-1 inhibition and thus hyperacetylation of p53, suggesting a predominantly p53-dependent, pro-apoptotic mechanism of action of resveratrol.

## 4 Discussion

The present study is dedicated to the crucial modulating role of HDAC Sirt-1 and p53 signaling by the bio-polyphenol resveratrol for its anti-tumor effect in human CRC cells, one of the most prevalent cancers. In this context, it has been reported that Sirt-1, as a NAD^+^-dependent deacetylase enzyme (41), deacetylates its certain substrates such as transcription factor p53, and thereby deactivates an important tumor suppressor protein in tumor cells (41, 42). Furthermore, a proven and significant up-regulation of Sirt-1 expression in many different tumors, including mouse adenocarcinomas, human colon cancer, breast cancer, squamous cell carcinoma, and prostate cancer cells, suggests Sirt-1 protein as a tumor mediator and therefore as a possible signaling target for the treatment of cancers (42–46).

The key observation of this work is resveratrol’s Sirt-1 inhibition at higher concentrations in HCT-116 CRC cells, thereby inducing pathways that are also down-stream of Sirt-1 signaling: (1) Resveratrol at higher concentrations (10-60µM) causes suppression of cell viability and plasticity as well as induction of apoptosis in human HCT-116 WT CRC cells, but not in human p53-deficient CRC cells (HCT-116 p53^-/-^). (2) In addition, resveratrol inhibits the expression of the protein Sirt-1 and its translocation to the nucleus, and in contrast activates acetylation and translocation of p53 to the nucleus in HCT-116 WT cells, as well as apoptosis. (3) Resveratrol significantly inhibited HCT-116 WT migration in a concentration-dependent manner but not in HCT-116 p53^-/-^ cells, similar to CRC HCT-116 WT cells transfected with Sirt-1-ASO. (4) Resveratrol at higher concentrations suppressed Sirt-1 expression, similar to the knockdown of Sirt-1 at the mRNA level, and led to up-regulation of acetylated p53, release of cytochrome C, and activation of caspase-3 in HCT-116 WT cells but not in HCT116 p53^-/-^ cells. (5) Finally, down-regulation of the deacetylase enzyme Sirt-1 by resveratrol promoted acetylation of its substrate p53, resulting in negative two-way crosstalk between Sirt-1 inhibition and thus hyperacetylation of p53, which was demonstrated by a co-immunoprecipitation assay. Thus, resveratrol exerts its pro-apoptotic mechanism of action, in part via a p53-dependent way.

The exact nature of the multiple functions of Sirt-1 signaling in carcinogenesis is still a matter of debate, as Sirt-1 can act as both a tumor enhancer and a repressor (42, 47). Sirtuins are a complex of NAD^+^-dependent deacetylases with correspondingly diverse metabolic properties. In mammals, there are seven sirtuins (Sirt-1 - Sirt-7), with Sirt-1, Sirt-6, and Sirt-7 occurring mainly in the nucleus, Sirt-3, Sirt-4, and Sirt-5 in mitochondria, and Sirt-2 in the cytosol. Originally, sirtuins were described as deacetylases, but a broader category of activities is now distinguished (24, 48, 49). Furthermore, Sirt-1 is the most intensively studied of all the sirtuins and it has been shown to deacetylate key histone residues on transcriptional control proteins and also several other proteins, including forkhead group of transcription factors O3 (FOXO3), tumor suppressor transcription factor p53, peroxisome proliferator-activated gamma receptor coactivator 1a (PGC-1a), and pro-inflammatory transcription factor nuclear factor (NF)-κB. Moreover, with the control of such important key proteins, Sirt-1 enzyme is able to control and regulate many important signaling cascades, such as DNA repair, glucose homeostasis, and apoptosis (49–51).

Previous studies from our own laboratory and others showed in healthy tissue cells that activation of Sirt-1 by resveratrol leads to deacetylation of the p53 signaling pathway and consequent inhibition of cell apoptosis and death (41, 52). In another previous work, we demonstrated resveratrol’s ability to mediate anti-proliferative and anti-metastatic function in CRC cells when applied in lower concentration (1-5µM) via Sirt-1-dependent deacetylation of pro-inflammatory and pro-cancerogenic transcription factor NF-κB (22).

The natural polyphenol resveratrol, which is normally occurring in the daily human diet from sources such as berries, grapes, peanuts, and more, has a broad panel of basic bioactive effects and is widely considered to be one of the most effective agents for modulating a number of signal transduction pathways involved in inflammation, cell plasticity and chronic diseases, including cancer, by inducing apoptosis and tumor cell death in a p53-dependent manner in various cancers (22, 53–58).

To test the hypothesis that resveratrol at higher concentrations in tumors has a down-regulatory effect on Sirt-1, thereby increasing its acetylating effect on p53 and inducing apoptosis, we treated HCT-116 WT and HCT-116 p53^-/-^cells with different concentrations of resveratrol (1-60µM) in this present paper and demonstrated a marked concentration-dependent down-regulation of proliferation, plasticity, migration, and apoptosis in HCT-116 WT but not in HCT-116 p53^-/-^ cells, and these results indicate p53-dependent resveratrol-induced apoptosis in CRC cells. Moreover, these results are consistent with preceding works showing a p53-dependent apoptosis induction by resveratrol in many different tumors, both *in vitro* and *in vivo* (53–56, 58–60). It is also of interest that some studies have shown that resveratrol at higher concentrations no longer activates Sirt-1 and its activation effect on Sirt-1 in the cell is even reversed, suggesting that the functional interaction between resveratrol and Sirt-1 in tumor cells depends on the concentration of resveratrol dose (61, 62).

We next demonstrated resveratrol’s inhibition of growth, plasticity, and migration as well as apoptosis initiation in a concentration-dependent manner in HCT-116 WT, but not in HCT-116 p53^-/-^ cells. The underlying signaling pathways were found to be significant in the activation of p53 and protein p21 and thereby inhibited cyclin D1, subsequent activation of caspase-3, and induction of apoptosis. Therefore, it is credible that resveratrol can dose-dependently curb CRC cell growth and migration by increasing the expression of potential p21 while decreasing the expression of cyclin D1 in HCT116 WT but not in HCT-116 p53^-/-^ cells. Accordingly, the protein p21 is the essential component of this regulatory system and this is consistent with other results, that acetylation of p53 leads to upregulation of p21 and inhibition of proliferation of CRC cells (57). These results are further in accordance with previous studies showing that increased acetylation and activation of the transcription factor p53 can significantly increase the release of cytochrome C from mitochondria and thus is known to increase cleavage and activation of caspase-3 (63–65).

Moreover, the expression of acetylated p53, p21, Bax, the release of cytochrome C, and cleavage of caspase-3 were significantly increased by silencing of Sirt-1 at the mRNA level in HCT-116, while at the same time the expression of cyclin D1 was down-regulated. This suggests a p53-related induction of caspase-3 activity to induce apoptosis of CRC cells promoted by resveratrol, proposing an underlying down-regulation of Sirt-1 signaling pathways. In line with other research, the disruption of Sirt-1 by NAD^+^ depletion causing an elevation in the expression of p53 and p21 in cancers and resulting in apoptosis has been previously reported (66, 67).

Notably, using a co-immunoprecipitation assay, we demonstrated that resveratrol at higher concentrations caused a negative reciprocal interplay between down-regulation of Sirt-1 and enhancement of p53 acetylation, thereby inducing p53-dependent apoptosis. Indeed, it suggests this pathway is one of the multiple signaling pathways of resveratrol that triggers cell death in CRC-TME.

## 5 Conclusion

These results demonstrate for the first time at higher resveratrol concentrations a negative reciprocal loop of down-regulation of Sirt-1 with simultaneous p53 acetylation, inhibiting plasticity and inducing apoptosis in CRC cells by promoting p53 as well as associated p21, Bax, cytochrome C, and cleaved caspase-3 signaling. With these findings, we contribute to clarifying a long-standing controversy and conclude that resveratrol may have p53-dependent pro-apoptotic as well as anti-plasticizing effects in CRC cells. Overall, this emphasizes resveratrol’s important anti-cancer possibilities and supports further research for its clinical utilization.

## Data availability statement

The original contributions presented in the study are included in the article/**Supplementary Material**. Further inquiries can be directed to the corresponding author/s.

## Author contributions

Design of working concept (AB and MS); conduct of investigations (AB and CB); methodology, validation, and analysis of results (AB and MS); writing, review, and editing (AB, CB, PS, and MS); project coordination (MS). Every author read and agreed on this manuscript before submission and publication.
