# Supplementary figures and images for: Resveratrol induces apoptosis by modulating the reciprocal crosstalk between p53 and Sirt-1 in the CRC tumor microenvironment
Source: Front Immunol. 2023 Jul 27;14:1225530. doi: 10.3389/fimmu.2023.1225530 (PMC10413256; doi:10.3389/fimmu.2023.1225530)

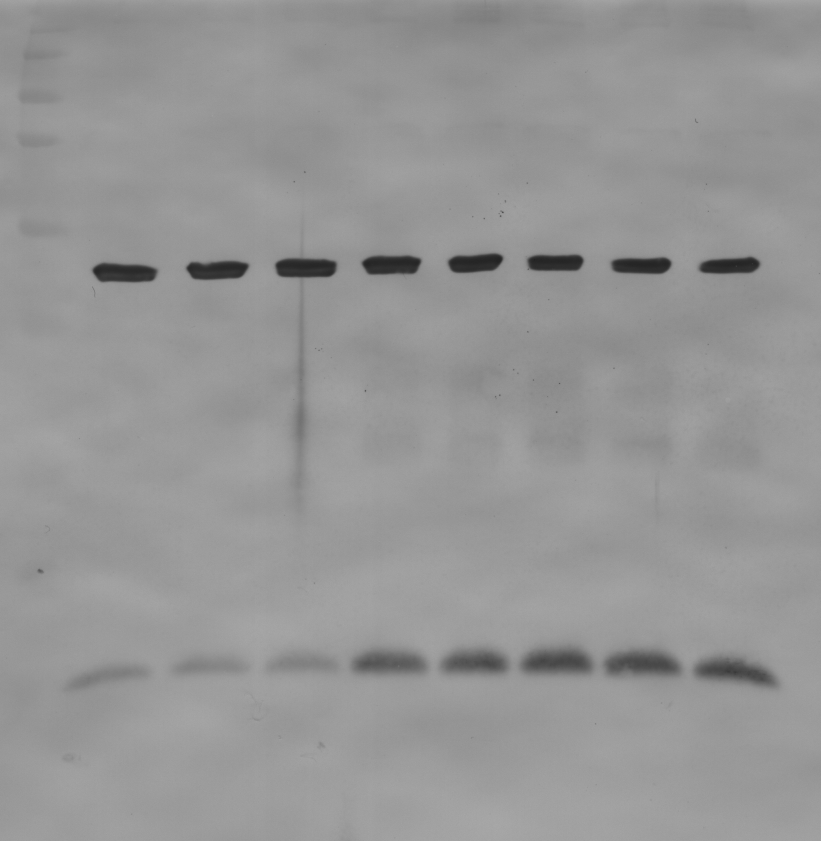

Supplement: Supplementary file 1 [file DataSheet_1.zip › Brockmueller et al-2023-Original-Figures/Figure 6/Figure 6A/Figure 6A-HCT-116WT-Caspase3-ß-Actinl-Original.tif]

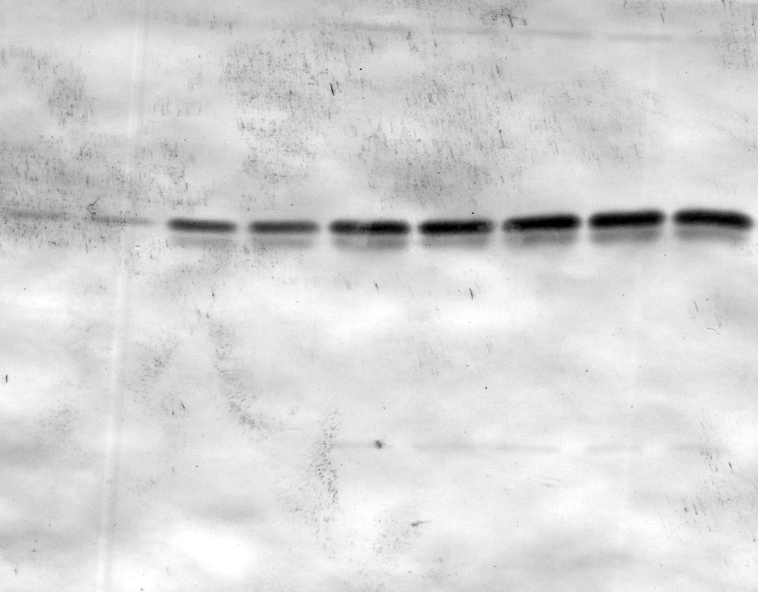

Supplement: Supplementary file 1 [file DataSheet_1.zip › Brockmueller et al-2023-Original-Figures/Figure 6/Figure 6A/Figure 6A-HCT-116WT-P53-Acetyl-Original.tif]

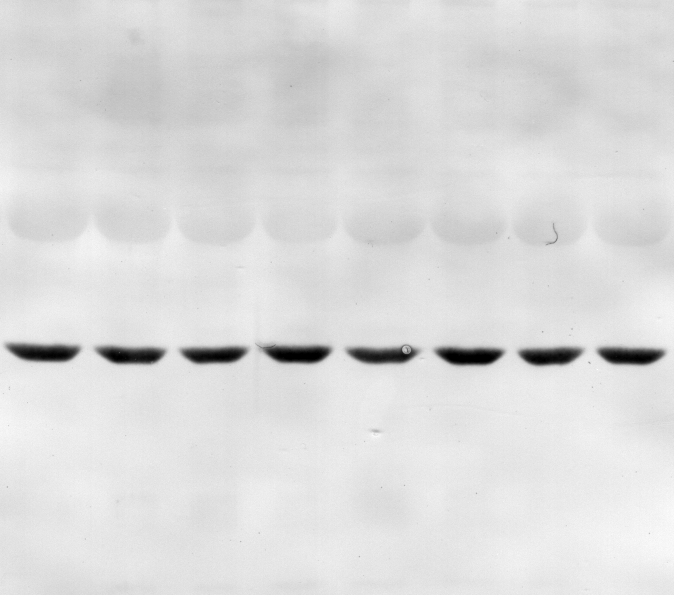

Supplement: Supplementary file 1 [file DataSheet_1.zip › Brockmueller et al-2023-Original-Figures/Figure 6/Figure 6A/Figure 6A-HCT-116WT-P53-Original.tif]

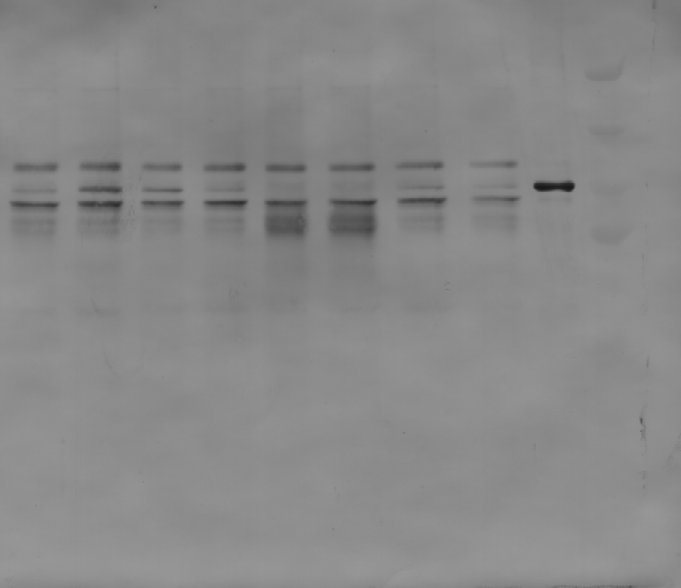

Supplement: Supplementary file 1 [file DataSheet_1.zip › Brockmueller et al-2023-Original-Figures/Figure 6/Figure 6A/Figure 6A-HCT-116-WT-SIRT1-Orig.tif]

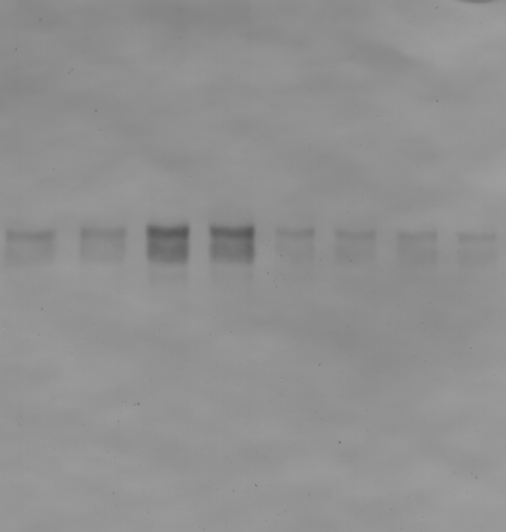

Supplement: Supplementary file 1 [file DataSheet_1.zip › Brockmueller et al-2023-Original-Figures/Figure 6/Figure 6B/Figure 6B-HCT-116WT-HCT-116p53Ko-acetyl-P53-Original.tif]

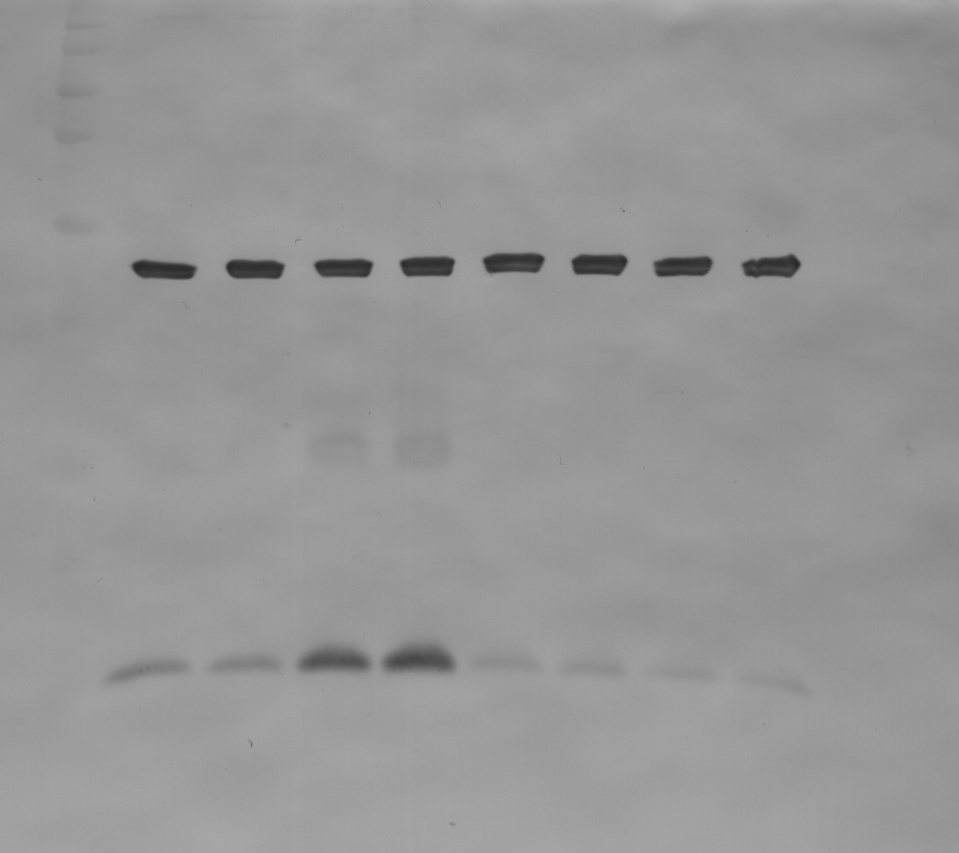

Supplement: Supplementary file 1 [file DataSheet_1.zip › Brockmueller et al-2023-Original-Figures/Figure 6/Figure 6B/Figure 6B-HCT-116WT-HCT-116p53Ko-Caspase3-ß-Actin-Origiginal.tif]

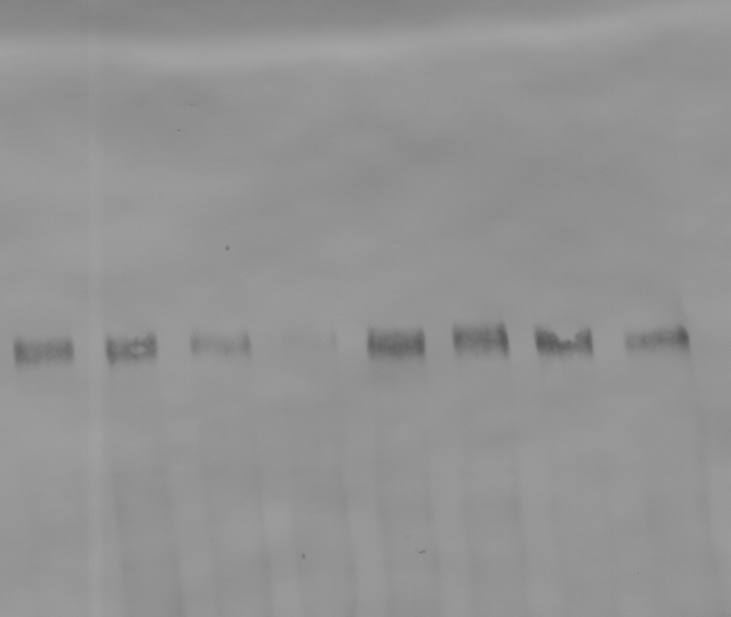

Supplement: Supplementary file 1 [file DataSheet_1.zip › Brockmueller et al-2023-Original-Figures/Figure 6/Figure 6B/Figure 6B-HCT-116WT-HCT-116p53Ko-CycD1-Original.tif]

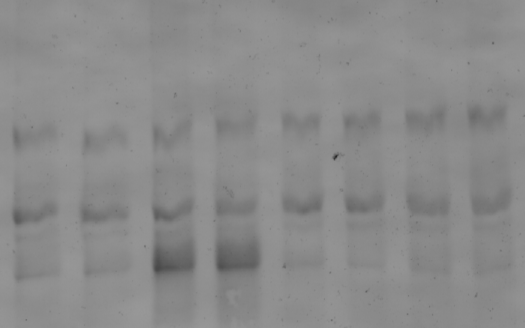

Supplement: Supplementary file 1 [file DataSheet_1.zip › Brockmueller et al-2023-Original-Figures/Figure 6/Figure 6B/Figure 6B-HCT-116WT-HCT-116p53Ko-P21-Original.tif]

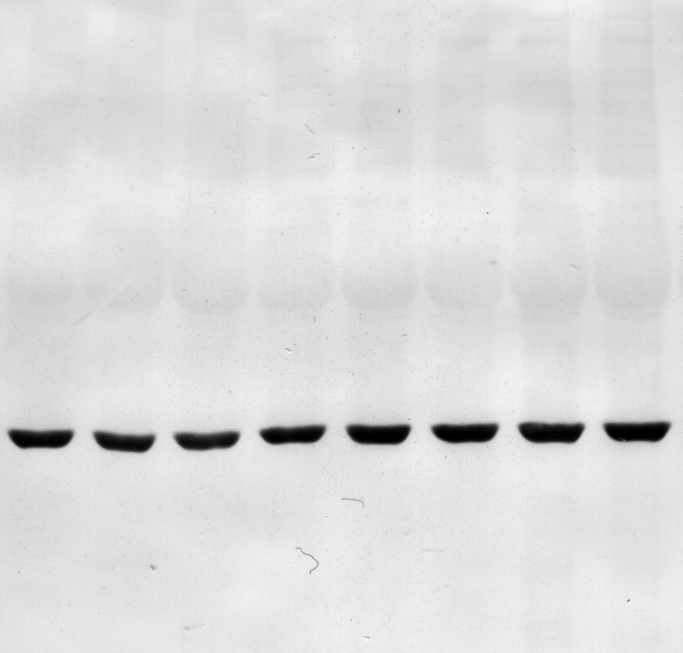

Supplement: Supplementary file 1 [file DataSheet_1.zip › Brockmueller et al-2023-Original-Figures/Figure 6/Figure 6B/Figure 6B-HCT-116WT-HCT-116p53Ko-ß-Actin-Original.tif]

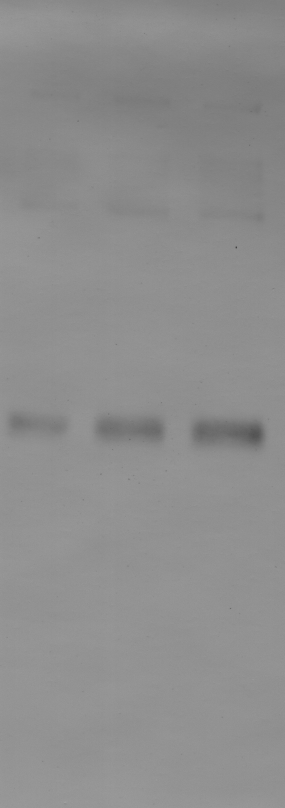

Supplement: Supplementary file 1 [file DataSheet_1.zip › Brockmueller et al-2023-Original-Figures/Figure 6/Figure 6C/Figure 6C-HCT-116WT-Sirt-1SO-ASO-acetylP53-Original.tif]

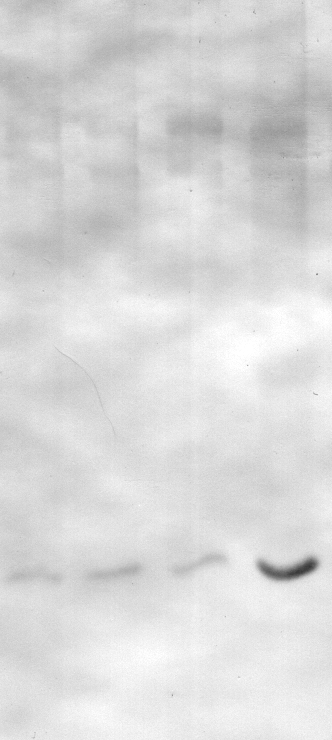

Supplement: Supplementary file 1 [file DataSheet_1.zip › Brockmueller et al-2023-Original-Figures/Figure 6/Figure 6C/Figure 6C-HCT-116WT-Sirt-1SO-ASO-Bax-Original.tif]

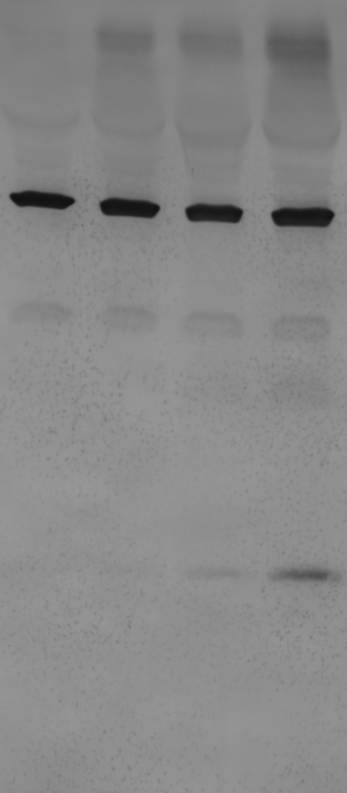

Supplement: Supplementary file 1 [file DataSheet_1.zip › Brockmueller et al-2023-Original-Figures/Figure 6/Figure 6C/Figure 6C-HCT-116WT-Sirt-1SO-ASO-Caspase3-ßactin-Original.tif]

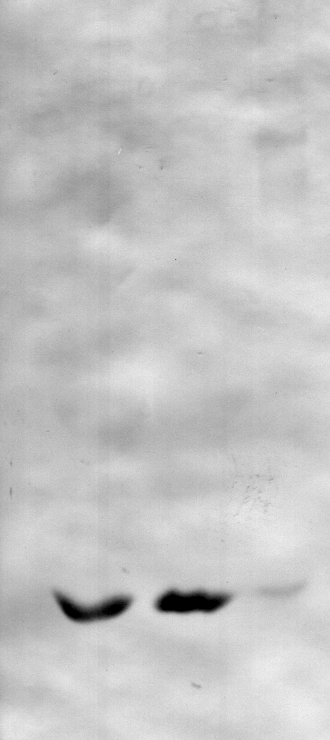

Supplement: Supplementary file 1 [file DataSheet_1.zip › Brockmueller et al-2023-Original-Figures/Figure 6/Figure 6C/Figure 6C-HCT-116WT-Sirt-1SO-ASO-cyclin D1-Original.tif]

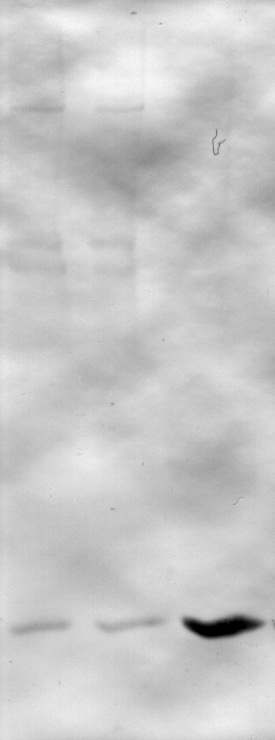

Supplement: Supplementary file 1 [file DataSheet_1.zip › Brockmueller et al-2023-Original-Figures/Figure 6/Figure 6C/Figure 6C-HCT-116WT-Sirt-1SO-ASO-cytochrome c-Originall.tif]

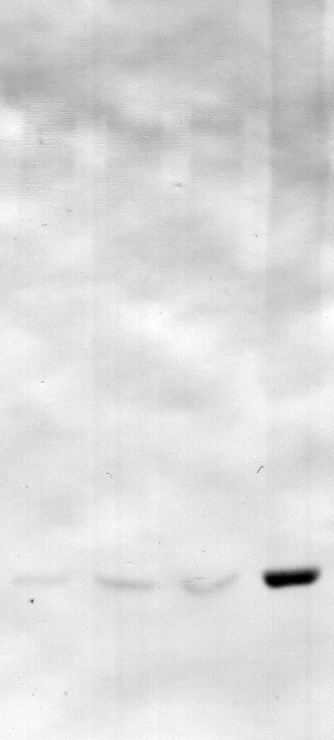

Supplement: Supplementary file 1 [file DataSheet_1.zip › Brockmueller et al-2023-Original-Figures/Figure 6/Figure 6C/Figure 6C-HCT-116WT-Sirt-1SO-ASO-p21-Original.tif]

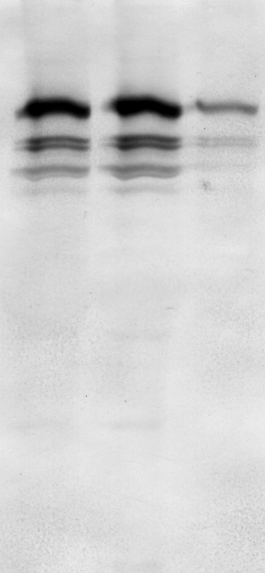

Supplement: Supplementary file 1 [file DataSheet_1.zip › Brockmueller et al-2023-Original-Figures/Figure 6/Figure 6C/Figure 6C-HCT-116WT-Sirt-1SO-ASO-Sirt-1-Original.tif]

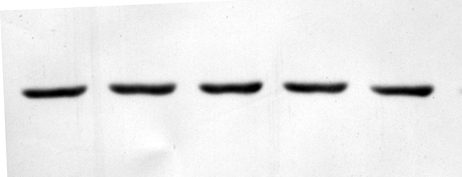

Supplement: Supplementary file 1 [file DataSheet_1.zip › Brockmueller et al-2023-Original-Figures/Figure 6/Figure 6C/Figure 6C-HCT-116WT-Sirt-1SO-ASO-ßActin-Original.tif]

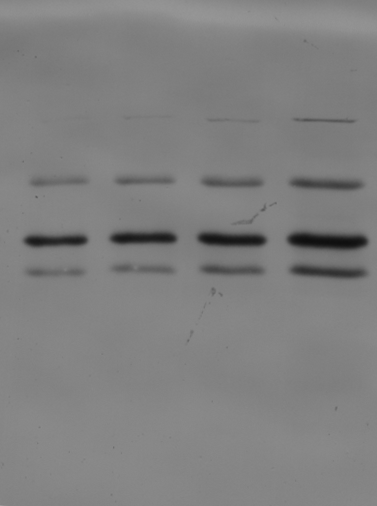

Supplement: Supplementary file 1 [file DataSheet_1.zip › Brockmueller et al-2023-Original-Figures/Figure 7/Figure 7A/Figure 7A-HCT-116WT-IP-acetyl-Lysin-WB-P53-FOXO3a-Original.tif]

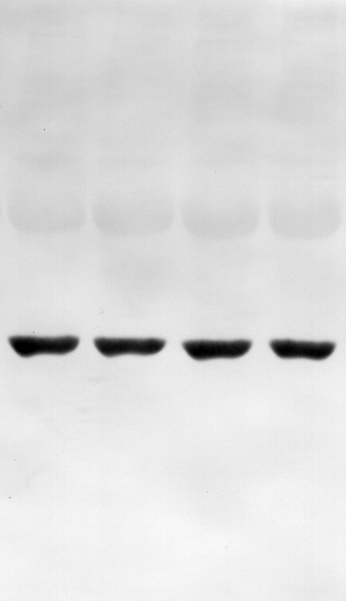

Supplement: Supplementary file 1 [file DataSheet_1.zip › Brockmueller et al-2023-Original-Figures/Figure 7/Figure 7A/Figure 7A-HCT-116WT-IP-acetyl-Lysin-WB-P53-Original.tif]

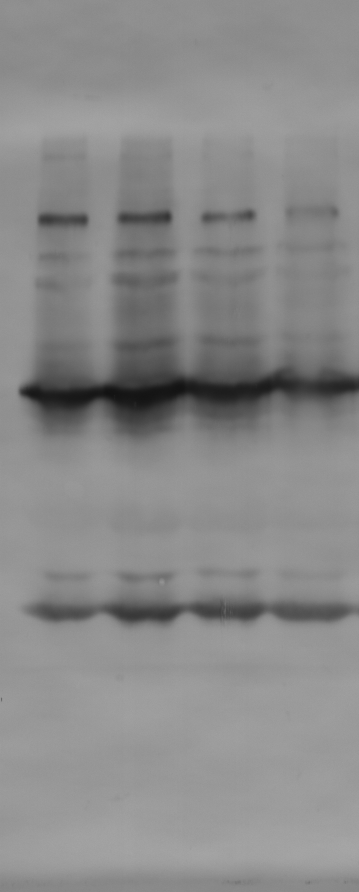

Supplement: Supplementary file 1 [file DataSheet_1.zip › Brockmueller et al-2023-Original-Figures/Figure 7/Figure 7B/Figure 7B-HCT-116WT-IP-p53-WB-Sirt1-Original.tif]

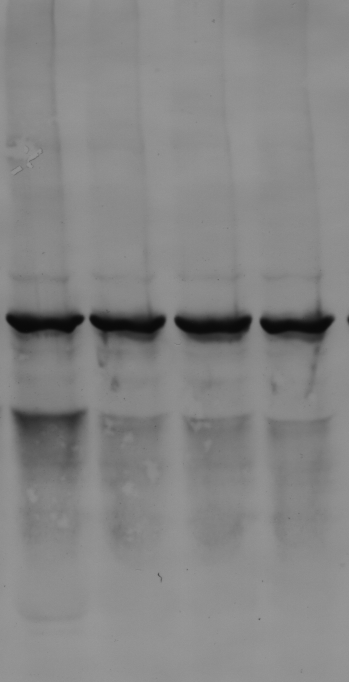

Supplement: Supplementary file 1 [file DataSheet_1.zip › Brockmueller et al-2023-Original-Figures/Figure 7/Figure 7B/Figure 7B-HCT-116WT-IP-Sirt1-WB-p53-Original.tif]

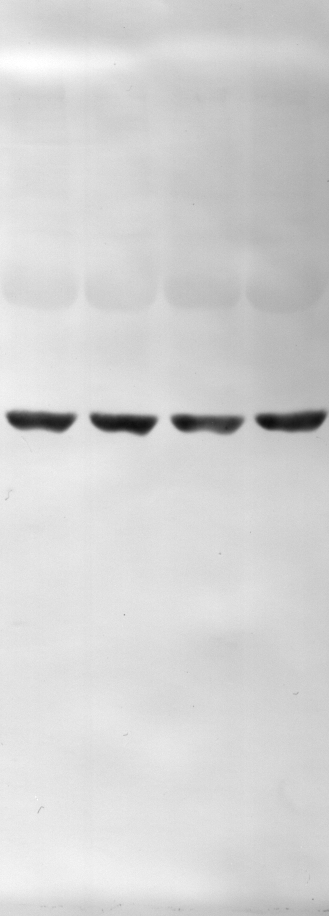

Supplement: Supplementary file 1 [file DataSheet_1.zip › Brockmueller et al-2023-Original-Figures/Figure 7/Figure 7B/Figure 7B-HCT-116WT-P53-Original.tif]
